# Quantification of wall shear stress using finite-element interpolations in multidimensional phase contrast MR data of the thoracic aorta

**DOI:** 10.1186/1532-429X-16-S1-P371

**Published:** 2014-01-16

**Authors:** Julio A Sotelo, Jesus Urbina, Ernesto Ortiz, Cristian Tejos, Israel Valverde, Daniel E Hurtado, Sergio Uribe

**Affiliations:** 1 Biomedical Imaging Center, Pontificia Universidad Católica de Chile, Santiago, Region Metropolitana, Chile; 2 Electrical Engineering, Pontificia Universidad Católica de Chile, Santiago, Region Metropolitana, Chile; 3 Radiology, School of Medicine, Pontificia Universidad Católica de Chile, Santiago, Region Metropolitana, Chile; 4 Structural Engineering, Pontificia Universidad Católica de Chile, Santiago, Region Metropolitana, Chile; 5 Biomedical Engineering Group, Pontificia Universidad Católica de Chile, Santiago, Region Metropolitana, Chile; 6 Pediatric Cardiology Unit, Hospital Universitario Virgen del Rocío, Sevilla, Spain; 7 Cardiovascular Physiopathology Laboratory, Biomedicine Institute of Seville, Hospital Universitario Virgen del Rocío/CSIC/Universidad de Sevilla, Sevilla, Spain

## Background

Different methods have been proposed to estimate Wall Shear Stress (WSS). Morgan and co-workers (J Thorac Cardiovasc Surg and Ann Biomed Eng 1998) used a finite difference scheme to quantify the WSS tensor. However, it is well known that finite-difference methods cannot effectively handle complex geometries, as those found in the cardiovascular system. To account for arbitrary cross-section shapes, Stalder et al., MRM 2008, used B-spline (BS) interpolations to smoothly describe the lumen contours. In this work, we propose and validate a new method for calculating the WSS distribution based on Finite-Element (FE) interpolations.

## Methods

The velocity field obtained at discrete locations from 2D and 3D CINE PC-MRI was interpolated using linear triangular FE. The shear stress tensor over the entire section cut was obtained from a global least-squares stress-projection method, from which the axial WSS vector was obtained. The proposed approach was benchmarked against a modified Poiseuille flow profile, and the robustness of the method was assessed by changing the level of resolution and noise. Additionally, we computed the WSS distribution in different aortic sections from a pulsatile aortic phantom, and from 5 healthy volunteers. In the aortic phantom and volunteers, 3D CINE PC-MRI flow data was acquired in 2D cutting planes in 5 with a spatial resolution 0.8 mm^2 ^and temporal resolution 37 ms. We have also compared our framework with a BS based method previously reported in the literature.

## Results

**Figure 1:**
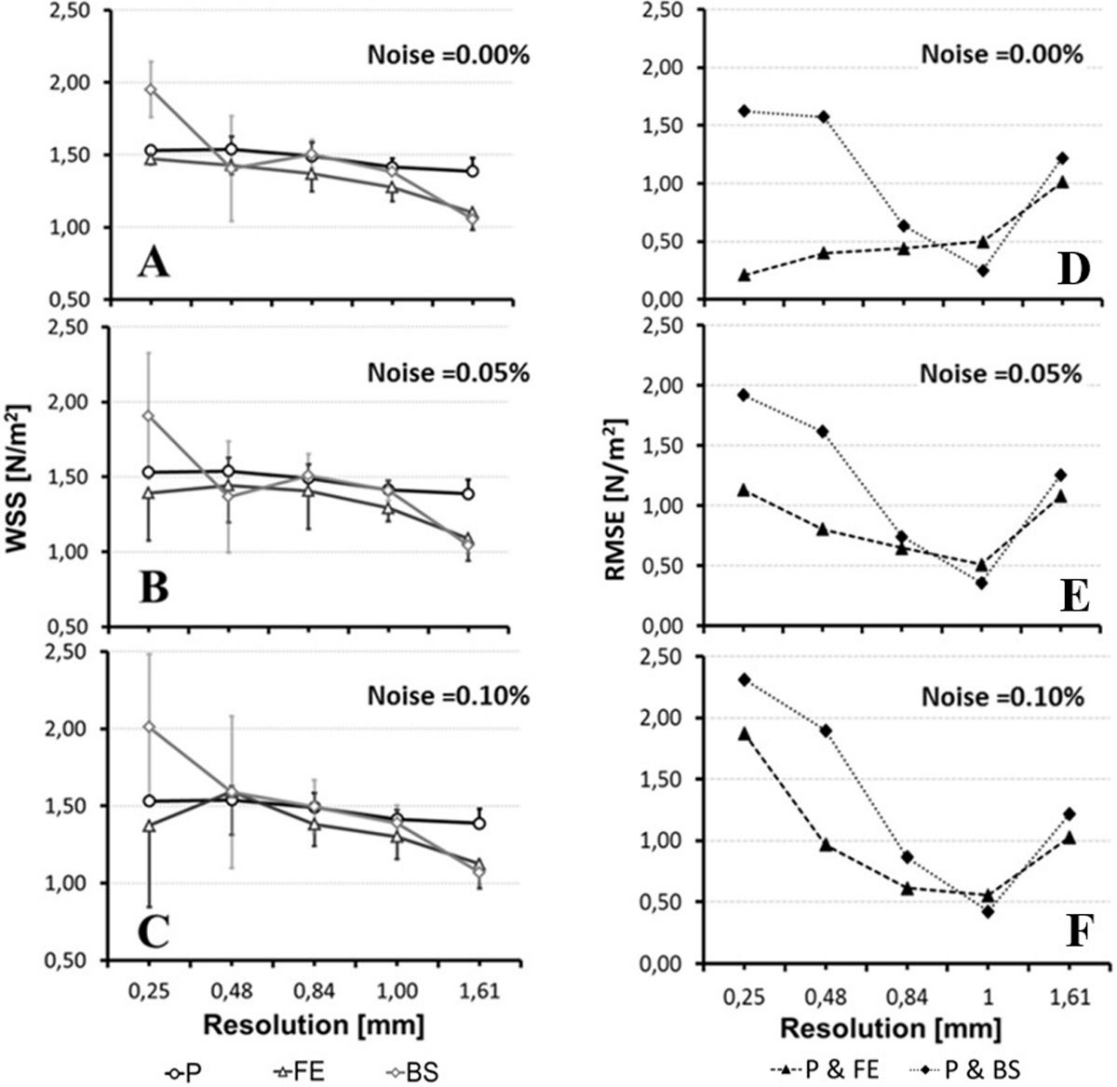
**WSS contour mean and standard deviation (left column), and root mean square error (RMSE) (right column) for a noise level of 0% of maximum velocity peak (A - D), 0.05% (B-E), and 0.1% (C - F). The methods analyzed were the modified Poiseuille flow (P), the proposed method (FE), and the B-Spline based method (BS)**.

**Figure 2:**
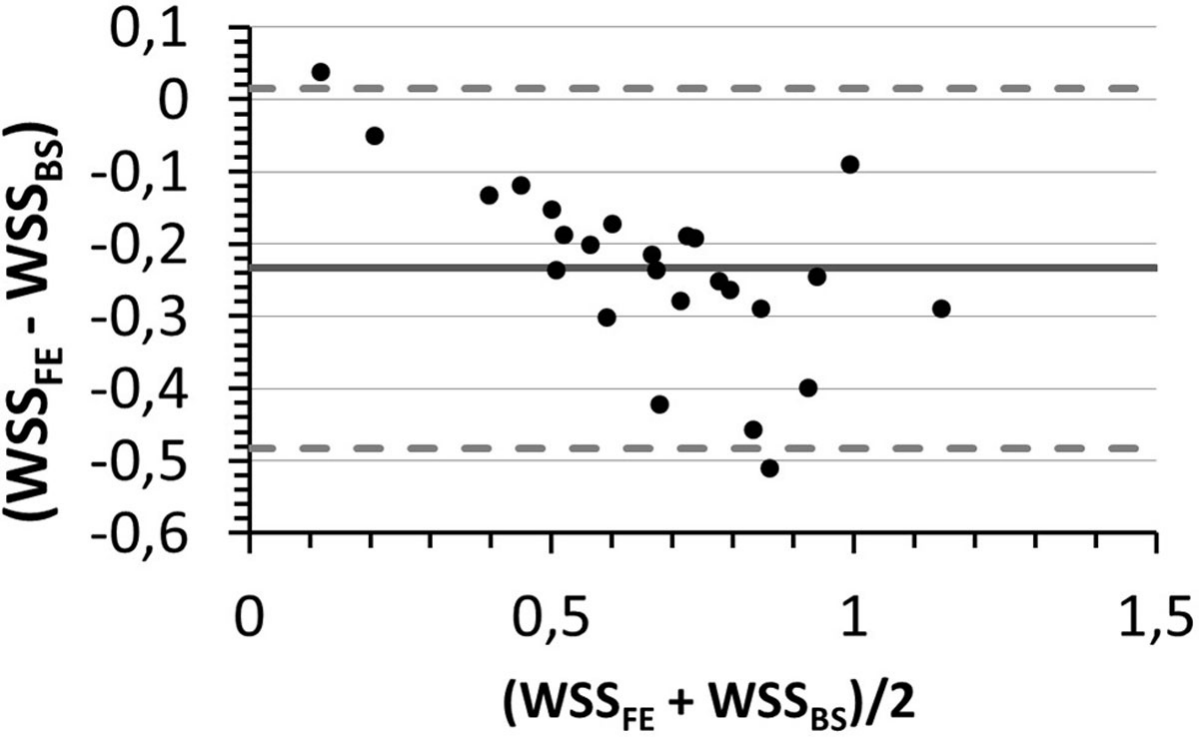
**Bland-Altman plot of cardiac phase averaged wall shear stress contour mean comparing the proposed method (FE) and the B-spline method (BS) from volunteer data**.

Our results showed that the local WSS values were in good agreement with the theoretical values obtained from the modified Poiseuille flow problem. The averaged WSS over the vessel contour showed a systematic, but negligible bias compared to the Poiseuille averaged WSS when subjected to different levels of noise and resolution, (see Figure 1). In contrast, the BS based method led to greater local differences, and in averaged WSS values that were largely affected by the level of noise and resolution. In volunteers, the cardiac cycle average value of the average WSS value was 0.21 ± 0.06 N/m^2^, whereas the BS based method yielded 0.45 ± 0.13 N/m^2^. The Bland-Altman plot (Figure 2) showed a systematic bias between both methods with a mean WSS difference of -0.2338 ± 0.2491 N/m^2^.

## Conclusions

In conclusion, we have developed a novel methodology to calculate WSS based on FE interpolations, which provides an excellent approximation of local WSS values, stability when subjected to noise and remarkable convergence properties as the pixel size is decreased.

## Funding

VRI # 44/2011 (Pontificia Universidad Católica de Chile), Anillo ACT 079 and FONDECYT #11100427 and #11121224. JS thanks CONICYT for scholarship for doctoral studies.

